# Normative Data and Reliability of the Test of Masticating and Swallowing Solids (TOMASS) in Healthy Brazilian Adults

**DOI:** 10.1111/1460-6984.70328

**Published:** 2026-09-23

**Authors:** Maria Eduarda Soares Arcaro, Amanda Bressanelli, Vanessa Brzoskowski do Santos, Alice Barbosa, Ed Bice, Maira Rozenfeld Olchik

**Affiliations:** ^1^ Postgraduate Program in Medical Science Universidade Federal do Rio Grande do Sul (UFRGS) Porto Alegre Rio Grande do Sul Brazil; ^2^ Undergraduate Program in Speech‐Language Pathology Universidade Federal do Rio Grande do Sul (UFRGS) Porto Alegre Rio Grande do Sul Brazil; ^3^ IOPI Medical, LLC Woodinville Washington USA; ^4^ Neurology Service Hospital de Clínicas de Porto Alegre (HCPA) Porto Alegre Rio Grande do Sul Brazil

**Keywords:** assessment, deglutition, mastication, solid, swallowing, TOMASS

## Abstract

**Introduction:**

The Test of Masticating and Swallowing Solids (TOMASS) is a standardized clinical assessment designed to quantify oral phase swallowing efficiency during solid food intake. Although TOMASS has been investigated in several countries, normative data for the Brazilian population are still lacking.

**Objective:**

To establish normative data and investigate the reliability of the Brazilian Portuguese version of TOMASS in healthy adults.

**Methods:**

This observational cross‐sectional study included 99 healthy adults stratified by age group. Individuals with dysphagia, head and neck cancer, neurological disorders, or gastrointestinal diseases were excluded. Participants underwent sociodemographic assessment, Montreal Cognitive Assessment (MoCA), oral structural evaluation, and TOMASS administration using a commercially available Cream Cracker biscuit available in Brazil. All assessments were video‐recorded for subsequent analysis. Test‐retest reliability was assessed through repeated administrations of TOMASS, and inter‐rater reliability was analysed using video recordings. The analysed variables included the number of bites, the number of masticatory cycles, the number of swallows, and total ingestion time. Statistical analyses included Student's *t*‐test, ANOVA with Bonferroni post hoc tests, Wilcoxon test, and ICC coefficient.

**Results:**

The sample included 99 healthy adults distributed across three age groups, with 54% women, a mean age of 50.2 (±16.3) years, mean education level of 12.4 (±4.1) years, and monthly household income equivalent to approximately two minimum wages (US$478 ± US$228). Significant differences were found between age groups in the number of masticatory cycles (*p* = 0.021), specifically between the 20–40 and 61+ age groups (*p* = 0.040). Significant differences were also observed in total task time (*p* < 0.001), with contrasts between the 20–40 and 61+ groups (*p* = 0.002), as well as between the 41–60 and 61+ groups (*p* = 0.013). Test‐retest reliability ranged from moderate to excellent, and inter‐rater reliability demonstrated good to excellent agreement across TOMASS variables.

**Conclusion:**

The Brazilian Portuguese version of TOMASS demonstrated satisfactory reliability and applicability for assessing mastication and swallowing performance in healthy Brazilian adults. The present study also provides normative reference data that may support future clinical and research applications of TOMASS in Brazil.

**WHAT THIS PAPER ADDS:**

*What is already known on the subject*

- The quantitative variables obtained through the TOMASS protocol contribute to the diagnostic accuracy of mastication abilities during a clinical swallowing examination (CSE), supporting more precise clinical decision‐making, particularly when compared with previously established normative values. Moreover, the quantitative data allow for monitoring biomechanical changes over time, whether due to natural processes such as aging, neurofunctional maturation, or spontaneous recovery, or attributed to the efficacy of specific therapeutic interventions.

*What this paper adds to existing knowledge*

- Normative TOMASS data for the Brazilian population may serve as clinical guidelines for assessing chewing and swallowing functions, given that the test is easily integrated into clinical evaluations due to its low cost and quick administration. These data may be especially relevant in contexts where socioeconomic, cultural, and physical barriers, a reality for a significant portion of the Brazilian population, still limit access to healthcare.

*What are the potential or actual clinical implications of this study?*

- The clinical implications of this study are significant for speech‐language pathologists in Brazil and other professionals involved in swallowing assessment and rehabilitation. The establishment of normative TOMASS data for the Brazilian population provides a valuable reference for identifying deviations in mastication performance across age and gender groups. Moreover, in low‐resource contexts and developing countries, a brief, easy‐to‐administer, and low‐cost protocol such as TOMASS is especially valuable, enabling equitable access to standardized assessment and monitoring of masticatory and swallowing functions.

## 1 Introduction

Adequate dentition is fundamental for mastication, an essential orofacial function that undergoes significant changes across the lifespan (Felício et al. 2012; Silva et al. 2017; Corassa et al. 2022). Physiologically, the stomatognathic system is responsible for the integrated processes of mastication and deglutition (Real et al. 2020; Pasinato et al. 2017). Mastication mechanically breaks down food through the coordinated action of the masticatory muscles, teeth, tongue, and central neural mechanisms (Pasinato et al. 2017).

Current assessments of mastication integrate both qualitative and quantitative approaches to evaluate chewing efficiency, functional performance, and jaw movement patterns. Kinetic and kinematic analyses, particularly through video recordings and electromyography, are commonly employed to capture the dynamics of jaw motion (Felício and Ferreira 2008). Additionally, advanced imaging modalities, such as computed tomography and magnetic resonance imaging—alongside chewing simulators, have been used to further characterize masticatory function (Fontijn‐Tekamp et al. 2000). When considering the current assessment methods, video‐based techniques have shown utility in analysing masticatory patterns (bilateral, unilateral, or incisal) and orofacial coordination. Despite the clinical applicability across diverse populations, the lack of methodological standardization remains a significant limitation (Felício and Ferreira 2008; Fontijn‐Tekamp et al. 2000).

Several instruments have been developed to assess mastication and swallowing performance, including self‐reported questionnaires, colorimetric chewing tests, and orofacial myofunctional protocols (Kosaka et al. 2016; Nokubi et al. 2010; Schimmel et al. 2007; Bueno et al. 2020; Medeiros et al. 2021). Self‐reported questionnaires, such as the Chewing Self‐Assessment Questionnaire (QAQM), provide information regarding patients’ perceptions of chewing ability across different food consistencies (Kosaka et al. 2016). In contrast, colorimetric tests using chewing gum or wax offer objective measures of masticatory efficiency based on colour homogeneity after chewing (Nokubi et al. 2010; Schimmel et al. 2007). Orofacial myofunctional protocols, such as MBGR and AMIOFE, combine qualitative and quantitative assessments of oral structures and functions, including chewing patterns, muscle performance, and compensatory behaviours (Bueno et al. 2020; Medeiros et al. 2021).

Although these instruments provide important clinical and functional information, some methods require laboratory or digital analysis, present limited international standardization, or have not yet been validated for the Brazilian population (Schimmel et al. 2007; Medeiros et al. 2021). In contrast, TOMASS provides a rapid, low‐cost, standardized, and objective assessment of solid bolus ingestion and has been increasingly used in international normative and clinical studies (Huckabee et al. 2018; Krishnamurthy et al. 2021; Sella‐Weiss 2023).

The Test of Mastication and Swallowing of Solids (TOMASS) is a standardized, specific, and quantitative tool developed to assess the functional efficiency of chewing and swallowing solid foods in a clinical setting (Huckabee et al. 2018). It is a quick test that uses a cracker as a standardized food, facilitating its use in different clinical contexts, including clinical assessments. It is suitable for both initial evaluations and therapeutic monitoring. Its validity and reliability have been confirmed in ten countries, including New Zealand, Australia, the United Kingdom, and the United States (Huckabee et al. 2018).

Previous studies have demonstrated that TOMASS performance may vary across populations and cultural contexts, reinforcing the importance of establishing local normative data. The original international normative study by Huckabee et al. (2018) included participants from New Zealand, Australia, the United Kingdom, and the United States, demonstrating differences in mastication and swallowing performance according to age and sex. Subsequently, Lamvik‐Gozdzikowska et al. (2019) reinforced the applicability of TOMASS as a quantitative measure of oral phase swallowing efficiency and highlighted the importance of validating the protocol in different populations. In India, Krishnamurthy et al. (2021) established normative TOMASS values for healthy adults and observed age‐related increases in mastication time and chewing cycles, emphasizing the influence of cultural eating habits and food characteristics on oral processing. Similarly, Hägglund et al. (2020) demonstrated that different commercially available crackers may significantly influence TOMASS performance, reinforcing the need for country‐specific normative data based on locally available foods.

Additional studies have expanded the applicability of TOMASS to different clinical and technological contexts. Todaro et al. (2021) investigated TOMASS performance in patients with dysphagia and demonstrated its usefulness in identifying alterations in oral phase efficiency. Sella‐Weiss (2023) reported associations between TOMASS performance, aging, and sex‐related variables, while Karlsson et al. (2023) demonstrated the feasibility of applying TOMASS through telemedicine assessments. Together, these findings suggest that TOMASS performance is influenced not only by biological factors such as age and sex, but also by cultural, socioeconomic, dietary, and methodological differences across populations, reinforcing the importance of establishing normative data for the Brazilian population.

Despite its wide international use and consolidated evidence of reliability, TOMASS has not yet been investigated in the Brazilian population, and normative reference values for Brazilian Portuguese speakers are currently unavailable. This represents a gap in the literature, considering the socioeconomic, cultural, dietary, and oral health differences that may influence mastication and swallowing performance across populations. Previous international studies have demonstrated that TOMASS performance may vary according to age, sex, type of test food, and regional characteristics, reinforcing the importance of establishing population‐specific normative data (Huckabee et al. 2018; Krishnamurthy et al. 2021; Hägglund et al. 2020). Brazil presents substantial regional inequalities in education, income, dietary habits, and access to oral healthcare, factors that may directly impact oral processing efficiency and swallowing behaviour. In addition, the availability of standardized and quantitative tools for assessing solid food ingestion remains limited in Brazilian clinical practice. Therefore, establishing Brazilian normative data for TOMASS may improve the clinical applicability of the protocol and contribute to a more accurate assessment of mastication and swallowing performance in the Brazilian population (Lamvik‐Gozdzikowska et al. 2019; Sella‐Weiss 2023).

### 1.1 Aims

To establish normative data and investigate the reliability of the Brazilian Portuguese version of TOMASS in healthy adults.

## 2 Methods

### 2.1 Study Design

This was a cross‐sectional observational study conducted with healthy Brazilian adults. The study aimed to establish normative data and investigate the reliability of the Brazilian Portuguese version of the Test of Masticating and Swallowing Solids (TOMASS).

The study was approved by the local Research Ethics Committee and all participants provided written informed consent before participation.

### 2.2 Participants

Participants were recruited from the general community through convenience sampling. Eligibility criteria included: age ≥18 years, being born in Brazil, native speaker of Brazilian Portuguese, and preserved cognitive ability sufficient to understand and follow the test instructions. Exclusion criteria included diagnosis of dysphagia, neurological diseases or injuries, gastrointestinal diseases, head and neck cancer, and inability to follow instructions during testing. All participants underwent cognitive screening using the Montreal Cognitive Assessment (MoCA), and individuals with scores below 26 points were excluded.

Participants were stratified into three age groups according to previous TOMASS normative studies 20–40 years, 41–60 years and 61 years and older.

#### 2.2.1 Sample Size Calculation

The sample size was estimated using the online PSS Health tool, considering a 95% confidence level and specific margins of error for the variables analysed. For the variable “number of bites,” an expected standard deviation of 1.5 and a margin of error of 0.5 were adopted, resulting in a minimum sample size of 38 participants. With the addition of 10% to account for contingencies such as losses and refusals, the number was adjusted to 43 individuals. Similarly, for the “number of chewing cycles,” with an estimated standard deviation of 30 and a margin of error of 10, the calculated sample size was 38 subjects, adjusted to 43 after the planned increment. For the “number of swallows,” with a standard deviation of 1.2 and a margin of error of 0.5, the minimum required sample was 25 participants, increasing to 28 when considering possible losses. The final sample of the study, composed of 99 individuals, exceeds the minimum sizes stipulated for the evaluated variables, providing greater robustness and reliability to the analyses conducted.

#### 2.2.2 Materials

For the clinical assessment of mastication and swallowing functions, a glass of water and a commercially available cracker widely distributed in Brazil, the Bauducco Cream Cracker, were used. Each cracker is composed of wheat flour enriched with iron and folic acid, vegetable fat, whey permeate, sugar, breadcrumbs, salt, emulsifier (soy lecithin), and chemical leavening agents (ammonium bicarbonate and sodium bicarbonate). Each unit weighs 4.2 g and measures 5.9 cm in height and 17.7 cm in width. An Apple iPhone 14 was used for video recording.

### 2.3 Translation and Cultural Adaptation

The original instructions of the Test of Mastication and Swallowing of Solids (TOMASS) underwent a systematic process of translation and cultural adaptation (Huckabee et al. 2018). Initially, two independent translations were carried out by bilingual researchers fluent in English and Portuguese, with experience in the field of speech‐language pathology. The translated versions were then compared and consolidated into a single consensus version that preserved the semantic and conceptual fidelity of the original text.

Subsequently, the consensus version was reviewed by the entire research team, who assessed the clarity, clinical relevance, and linguistic appropriateness of the instructions for the Brazilian target population. Minor linguistic adaptations were performed while preserving the conceptual structure of the original protocol proposed by Huckabee et al. (2018). It is important to note that, in the present study, additional oral health variables not originally included in the TOMASS protocol were recorded, including the number of natural teeth, the presence of dental prostheses, and the use of orthodontic appliances.

### 2.4 Procedures

Sociodemographic data, including age, sex, education level, and monthly household income, were collected through an interview before administering the TOMASS. Education level was categorized according to years of formal schooling, and income was classified based on the number of Brazilian minimum wages. All participants underwent cognitive screening using the MoCA.

Participants were instructed to remain comfortably seated in a chair and subsequently provided their sociodemographic data. Participants were then provided with a glass of water, two crackers, and a napkin. They were instructed not to speak during the task and received the following command: “Eat the cracker as quickly and comfortably as possible, and when you finish, say your name out loud.” Timing began when the cracker passed the participant's lips and ended when the participant verbally stated their name after swallowing.

During the observation of the test, the following variables were recorded:

1. Number of bites: determined by the number of pieces into which the cracker was broken and ingested.
2. Number of swallows: counted by observing the thyroid cartilage movement.
3. Number of masticatory cycles: observed by tracking jaw movements.
4. Total ingestion time: measured from the moment the cracker passed the lips to when the participant said their name.

Following the completion of each test, and in accordance with the procedures described in the original TOMASS protocol, a detailed inspection of the oral cavity was performed to identify the presence of food residue (Huckabee et al. 2018). Given that the events under investigation occur rapidly and require high observational precision, the entire procedure was video recorded to enable subsequent analysis by an independent evaluator, ensuring accuracy in the quantification of the variables of interest, the number of chewing cycles, and the number of swallows.

### 2.5 Reliability Analysis

In the first stage, a single examiner conducted the test–retest in person on the same day, strictly following the same protocol and instructions for all participants. All assessments were video recorded and independently analysed by a second examiner blinded to the first evaluation in order to determine inter‐rater agreement. The agreement between examiners was calculated using the Intraclass Correlation Coefficients (ICC). The inter‐rater agreement was considered excellent, with an ICC index ≥0.90, indicating high consistency and reproducibility of the assessments.

This study focused on establishing normative data and investigating reliability measures of the Brazilian Portuguese version of TOMASS rather than performing formal construction or criterion validity testing.

### 2.6 Statistical Analysis

Descriptive statistics were used to characterize the sample, including absolute and relative frequencies, means and standard deviations, and medians with interquartile ranges, when appropriate. The independent variables analysed included sex, age, education level, household income, MoCA score, number of teeth, use of orthodontic appliances, and presence of dental prostheses. Data distribution was assessed using the Kolmogorov–Smirnov and Shapiro–Wilk tests. The choice of statistical analyses was based on the distribution and characteristics of the variables.

Primary analyses included comparisons of TOMASS variables across age groups and reliability analyses, including test‐retest reliability and inter‐rater agreement. Comparisons among age groups were performed using one‐way analysis of variance (ANOVA), followed by Bonferroni post hoc tests when appropriate.

Exploratory analyses included comparisons between sexes and descriptive evaluation of dental characteristics, including the number of teeth, use of dental prostheses, and use of orthodontic appliances. Comparisons between sexes within each age group were performed using Student's *t*‐test.

Test‐retest comparisons were analysed using the non‐parametric Wilcoxon signed‐rank test. Inter‐rater reliability for continuous TOMASS variables was assessed using the Intraclass Correlation Coefficient (ICC), considered appropriate for quantitative continuous measures. Statistical significance was set at *p* < 0.05. All statistical analyses were performed using SPSS software, version 22.0.

## 3 Results

A total of 99 healthy adults participated in the study and were distributed across three age groups: 20–40 years (*n* = 37), 41–60 years (*n* = 29), and 61 years and older (*n* = 33). Sociodemographic characteristics are presented in Table 1. Mean years of education ranged from 9.45 to 12.79 years, with the highest values observed in the 41–60 age group. Dental prosthesis use was absent in the 20–40 age group and was observed in 66.7% of participants aged 61 years or older. MoCA scores showed a progressive reduction across age groups

**TABLE 1:** Sociodemographic data of the sample stratified into three age groups.

	20–40 (*n* = 37)		41–60 (*n* = 29)		61 + (*n* = 33)	
Sex	Female	26 (70.3%)	Female	14 (48.3%)	Female	14 (42.4%)
	Male	11 (29.7%)	Male	15 (51.7%)	Male	19 (66.7%)
Age (years)	20.62 (±5.4)		50.62 (±5.44)		70.94 (±6.08)	
Education (years)	10.41 (±6.0)		12.79 (±5.05)		9.45 (±4.28)	
Household income	3 (2–6.5)		3 (1.75–4)		2 (1.13–3)	
MoCA	28.0 (±1.96)		24.86 (±3.63)		22.67 (±4.34)	
Number of teeth	20–32		20–32		15–25	
Orthodontic appliance	No	32 (86.5%)	No	29 (100%)	No	33 (100%)
	Upper	2 (5.4%)	Upper	—	Upper	—
	Lower	1 (2.7%)	Lower	—	Lower	—
	Upper /Lower	2 (5.4%)	Upper /Lower	—	Upper /Lower	—
Dental prosthesis	No	37 (100%)	No	23 (79.3%)	No	11 (33.3%)
	Yes	—	Yes	6 (20.7%)	Yes	22 (66.7%)

*Notes*: Household income: corresponds to the number of minimum wages (1 minimum wage = R$1518.00). Data are presented as mean/standard deviation and percentage, except for household income (median and interquartile range).

Abbreviation: MoCA, Montreal Cognitive Assessment.

Table 2 presents the descriptive TOMASS data according to age group and sex during the test and retest sessions. Overall, similar performance was observed between test and retest sessions across all age groups for the number of bites, masticatory cycles, number of swallows, and total ingestion time.

**TABLE 2:** Mean values of TOMASS variables by sex in each age group in the test‐retest.

		Test				Retest			
Age	Sex	Number of bites	Number of chewing cycles	Number of swallows	Total time (sec)	Number of bites	Number of chewing cycles	Number of swallows	Total time (sec))
**20–40**	Female	3.23 (±1.1)	50.69 (±10.72)	2.88 (±0.99)	47.27 (±11.5)	3.46 (±1.24)	49.42 (±12.17)	3.0 (±1.32)	46.0 (±13.86)
	Male	3.0 (±1.18)	49.91 (±17.53)	2.18 (±1.16)	50.27 (±17.48)	2.91 (±1.44)	47.0 (±17.26)	2.0 (±1.18)	45.09 (±19.95)
**41–60**	Female	3.5 (±1.22)	54.36 (±28.31)	2.43 (±1.39)	59.86 (±23.06)	4.07 (±1.63)	53.21 (±24.76)	2.79 (±1.18)	55.86 (±21.64)
	Male	3.13 (±1.18)	43.43 (±10.35)	1.57 (±0.64)	38.6 (±10.81)	3.33 (±1.04)	47.43 (±9.95)	1.79 (±0.57)	40.6 (±10.39)
**61+**	Female	3.14 (±1.02)	55.86 (±14.64)	2.14 (±0.86)	58.0 (±17.18)	3.71 (±1.85)	60.21 (±25.82)	2.71 (±0.99)	66.5 (±31.05)
	Male	3.11 (±1.37)	71.58 (±39.46)	2.16 (±0.68)	64.89 (±30.03)	3.32 (±1.79)	70.05 (±52.52)	2.28 (±0.82)	66.68 (±40.66)

*Note*: No.: number.

Abbreviation: Sec, seconds.

**FIGURE 1:**
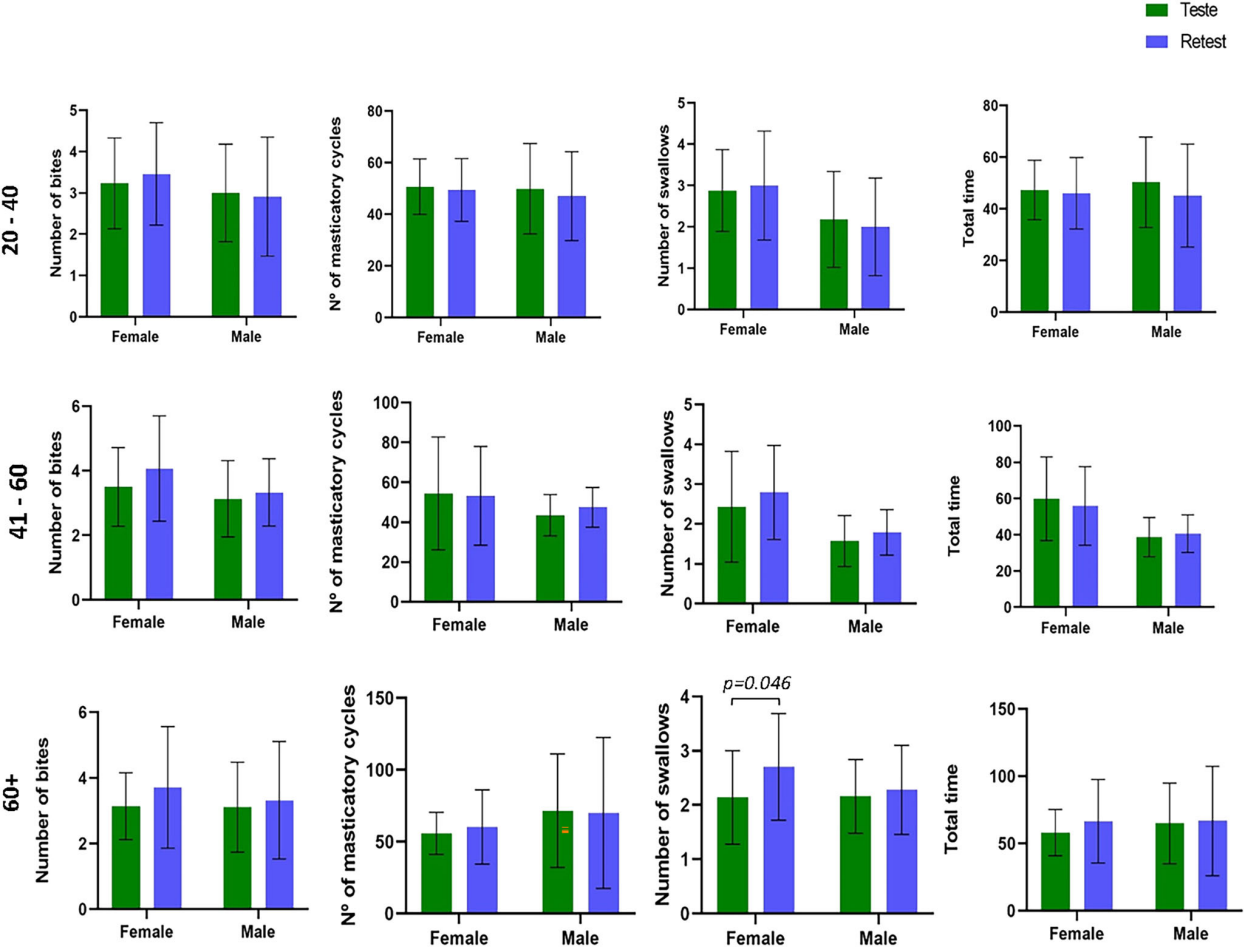
Test‐retest comparison by sex in each group. .

Figure 1 presents the test‐retest comparisons according to sex and age group. Overall, no statistically significant differences were observed between test and retest measures across the analysed TOMASS variables, indicating good test‐retest reliability. The only significant difference identified was for the number of swallows among females aged 61 years and older (*p* = 0.046).

**FIGURE 2:**
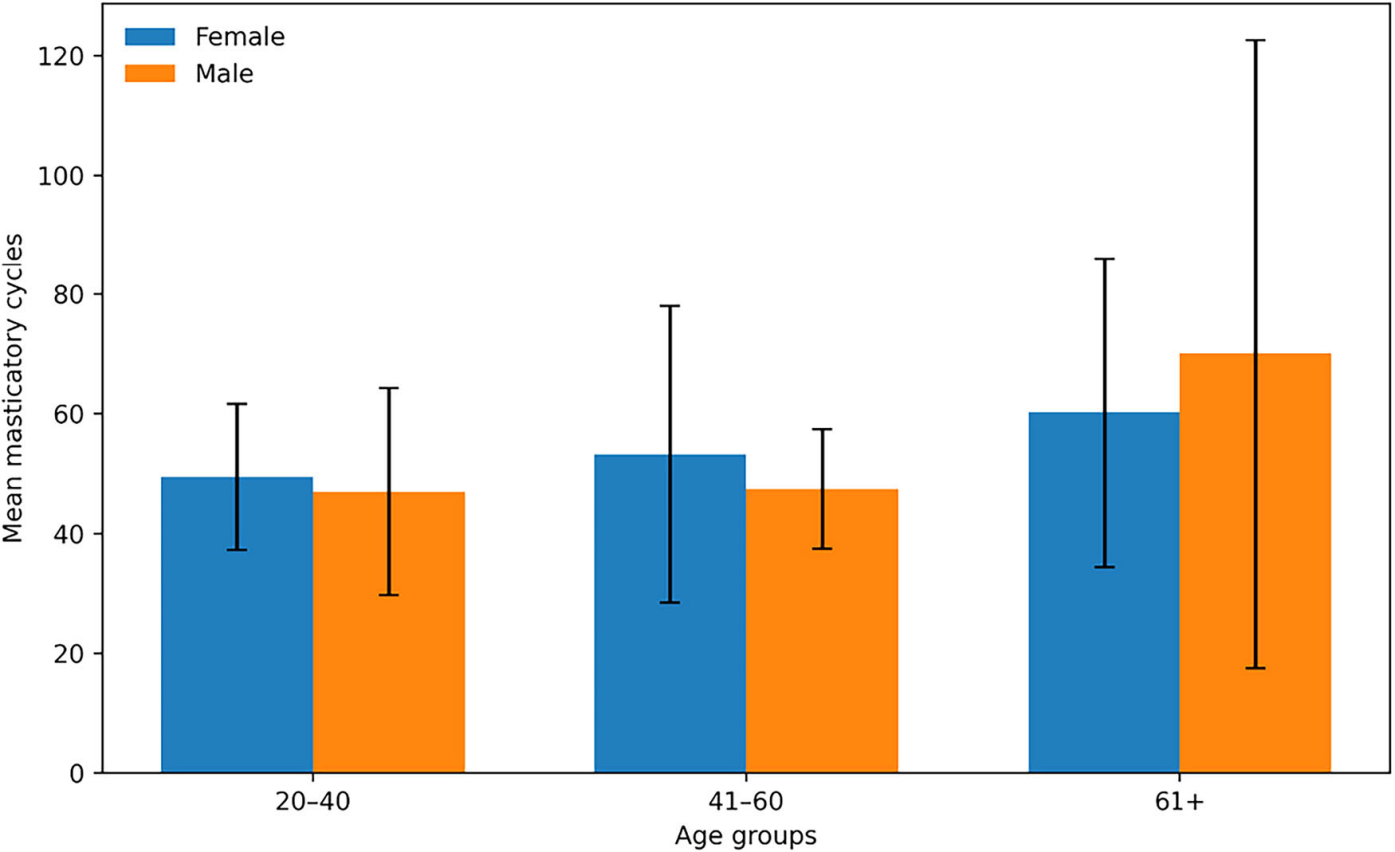
Comparison between sexes in each age group.

Figure 2 shows comparisons between sexes within each age group using retest values revealed no statistically significant differences for any of the analysed TOMASS variables.

**FIGURE 3:**
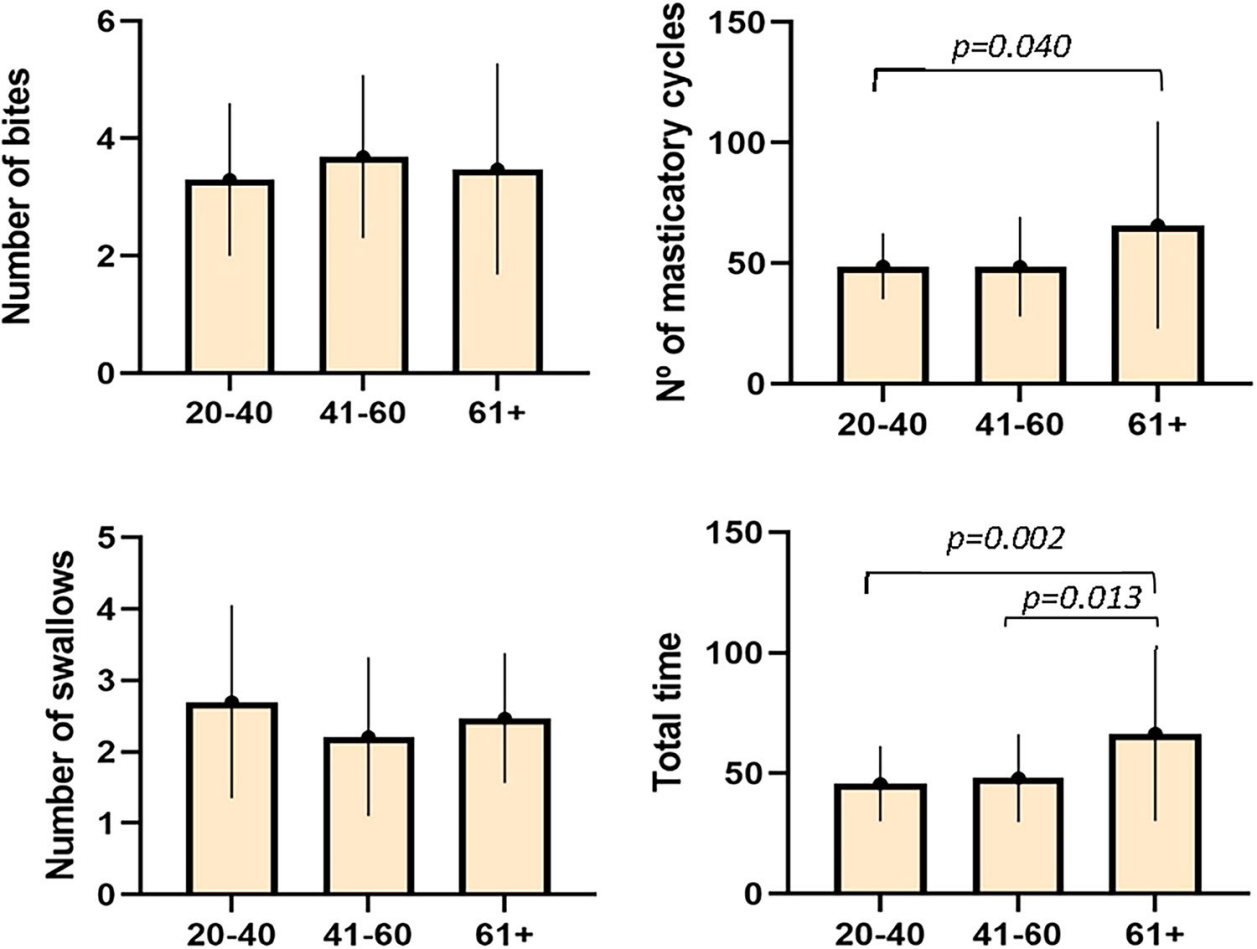
Comparison between groups for the TOMASS variables.

Figure 3 comparisons among age groups using retest values demonstrated significant differences for the number of masticatory cycles (*p* = 0.021) and total ingestion time (*p* < 0.001). Participants aged 61 years and older showed poorer TOMASS performance, with higher numbers of masticatory cycles and longer total ingestion times compared with younger groups. Post hoc analyses confirmed significant differences between the 20–40 and 61+ groups for masticatory cycles (*p* = 0.040), and between the older group and both younger groups for total ingestion time.

Table 3 summarizes the primary analyses across age groups, including descriptive statistics, *p* values, and effect sizes. Moderate effect sizes were observed for the number of masticatory cycles (*η* ^2^ = 0.07), while total task time demonstrated the largest effect size (*η* ^2^ = 0.13), indicating a greater influence of age on overall TOMASS performance.

**TABLE 3:** Summary of primary analyses across age groups, including effect sizes.

Variable	20–40 years Mean ± SD	41–60 years Mean ± SD	61+ years Mean ± SD	*p* value	Effect size (*η* ^2^)
Number of bites	3.30 ± 1.31	3.69 ± 1.39	3.48 ± 1.80	0.581	0.01
Number of masticatory cycles	48.70 ± 13.67	50.32 ± 18.75	65.88 ± 42.98	0.026	0.07
Number of swallows	2.70 ± 1.35	2.29 ± 1.05	2.47 ± 0.92	0.338	0.02
Total time (seconds)	45.73 ± 15.63	47.97 ± 18.21	66.61 ± 36.36	0.001	0.13

*Note*: η^2^: eta squared effect size.

Table 4 presents the agreement between raters, and the test‐retest analyses demonstrated good to excellent reliability across all variables of the TOMASS questionnaire in the Brazilian Portuguese version.

**TABLE 4:** Inter‐rater reliability for TOMASS variables.

Variable	ICC	95% CI	Interpretation
Number of bites	0.89	0.83–0.93	Good
Number of masticatory cycles	0.92	0.88–0.95	Excellent
Number of swallows	0.85	0.78–0.91	Good
Total time	0.94	0.91–0.97	Excellent

Abbreviation: ICC: intraclass correlation coefficient.

Regarding dental characteristics, participants aged 61 years and older presented fewer teeth and a higher frequency of dental prosthesis use compared with younger groups.

## 4 Discussion

The present study established normative data and investigated the reliability of the Brazilian Portuguese version of TOMASS in healthy adults across different age groups. Overall, the findings demonstrated satisfactory test‐retest and inter‐rater reliability, supporting the consistency and reproducibility of TOMASS measurements in the Brazilian population. These findings are consistent with the original TOMASS study conducted by Huckabee et al. (2018), as well as with the Indian adaptation described by Kothari et al. (2021). The satisfactory reliability observed in the present study reinforces the potential applicability of TOMASS in Brazilian clinical and research settings, particularly because the protocol provides objective and reproducible measures of solid bolus ingestion (Huckabee et al. 2018; Kothari et al. 2021).

The establishment of normative data for the Brazilian Portuguese version of TOMASS may contribute to the clinical assessment of mastication and swallowing in both healthy and clinical populations. The need for population‐specific TOMASS normative data has been highlighted in previous international studies, since cultural, dietary, and oral health characteristics may influence solid bolus ingestion performance (Huckabee et al. 2018; Krishnamurthy et al. 2021; Sella‐Weiss 2023).

The protocol is simple, low‐cost, and easily applicable in clinical practice, allowing objective assessment of solid bolus ingestion (Huckabee et al. 2018). In addition, the availability of normative TOMASS data for Brazilian Portuguese speakers represents an important step for future investigations involving dysphagia and neurological populations (Kothari et al. 2021).

The use of standardized protocols such as TOMASS may facilitate the identification of alterations in oral preparatory and oral transit phases during solid food intake. In clinical practice, objective measures related to chewing and swallowing performance may support the assessment and monitoring of individuals with suspected dysphagia and other neurogenic or age‐related swallowing disorders (Matsuo and Palmer 2009; Huckabee et al. 2018).

Age‐related differences were identified mainly for the number of masticatory cycles and total ingestion time. Participants aged 61 years and older demonstrated higher numbers of masticatory cycles and longer total ingestion times compared with younger adults. Similar findings have been reported in previous TOMASS studies and are consistent with the physiological changes associated with aging (Huckabee et al. 2018; Kothari et al. 2021). Reductions in oromotor efficiency, tongue strength, muscle performance, and salivary flow may contribute to slower oral processing and increased chewing effort in older adults (Türp et al. 2016; Gonçalves et al. 2021; Affoo et al. 2015). Age‐related changes in mastication and swallowing are multifactorial and involve neuromuscular, sensory, and functional adaptations. Older adults frequently demonstrate compensatory oral processing strategies aimed at maintaining swallowing safety and efficiency during solid bolus ingestion. These adaptations may contribute to the increased number of masticatory cycles and longer ingestion times observed in the present study (Affoo et al. 2015; Yoshikawa et al. 2011; Gonçalves et al. 2021).

The longer ingestion times and higher number of masticatory cycles observed among older adults may also reflect compensatory adaptations associated with aging. Previous studies suggest that older individuals often require greater oral processing effort to maintain safe and efficient swallowing, particularly during solid food intake (Matsuo and Palmer 2009; Yoshikawa et al. 2011).

No statistically significant differences were observed between sexes across the analysed TOMASS variables. Although variations between males and females have been described in previous studies, the present findings do not support substantial sex‐related differences in TOMASS performance within this sample (Park and Shin 2015; Tamura and Shiga 2014).

Older participants also presented fewer teeth and a higher frequency of dental prosthesis use compared with younger groups. Although these oral health characteristics may influence mastication performance, the present study was not specifically designed to investigate the association between dental status and TOMASS variables. Therefore, causal interpretations regarding the effects of tooth loss or prosthesis use should be made with caution (Gotfredsen and Walls 2008; Huang et al. 2021).

The specialized literature on dental arch morphology indicates that a reduced number of natural teeth is associated with significant functional impairments (Gerritsen et al. 2010). Participants were classified into four categories based on their dentition status: edentulism (0 teeth), severe tooth loss (1–9 teeth), moderate tooth loss (10–19 teeth), and complete dentition (20–32 teeth). This cutoff is based on established literature, which indicates that masticatory efficiency declines markedly below 20 teeth, particularly when fewer than 9–10 occlusal pairs are present (Dai et al. 2022; Chalub et al. 2016; Gotfredsen and Walls 2008).

The findings of the present study are consistent with previous international TOMASS investigations demonstrating that mastication and swallowing performance may vary according to population characteristics, eating habits, oral health conditions, and methodological differences. Huckabee et al. (2018), in the original multinational normative study, reported significant effects of age on TOMASS performance, particularly regarding mastication cycles and total ingestion time, findings that were also observed in the present Brazilian sample. Similarly, Krishnamurthy et al. (2021), in the Indian population, identified longer chewing times and increased mastication cycles among older adults, suggesting that aging consistently influences oral processing efficiency across different cultural contexts.

International studies have also demonstrated that TOMASS performance may be influenced by characteristics of the test food itself. Hägglund et al. (2020) reported variations in TOMASS outcomes according to the type of commercially available cracker used in Scandinavian populations, reinforcing the importance of establishing country‐specific normative values using foods available in local markets. In Brazil, cultural eating habits, socioeconomic disparities, oral health conditions, and differences in access to dental care may also contribute to variations in mastication performance, supporting the relevance of locally derived normative data.

In addition, previous studies have expanded TOMASS applicability to different clinical and technological settings. Todaro et al. (2021) demonstrated the utility of TOMASS in patients with dysphagia, while Karlsson et al. (2023) reported satisfactory agreement between face‐to‐face and telemedicine assessments. Together, these studies reinforce the clinical applicability of TOMASS and support the importance of establishing reliable normative reference values for Brazilian Portuguese speakers.

The age stratification adopted in the present study differed from previous TOMASS normative studies because it was designed to reflect the distribution of the recruited Brazilian sample and to ensure adequate representation across different stages of adulthood and aging (Huckabee et al. 2018; Krishnamurthy et al. 2021; Sella‐Weiss 2023). In addition, an unequal distribution between sexes was observed across age groups, particularly in the younger group, which presented a predominance of female participants. This distribution may reflect recruitment characteristics and participant availability during the data collection period, which is commonly observed in convenience sampling approaches used in observational studies (Sedgwick 2013).

Some limitations should be considered. First, participants were recruited from a single region of Brazil, which may limit the generalizability of the findings to other Brazilian populations. Second, the sample presented an unequal sex distribution across age groups. Third, the study primarily focused on reliability analyses and normative data, without a formal assessment of construct or criterion validity (Mokkink et al. 2010). Finally, although the sample size was adequate for the proposed analyses, larger multicenter studies may provide more representative normative data for the Brazilian population (Huckabee et al. 2018; Krishnamurthy et al. 2021).

Future studies should include participants from different Brazilian regions and investigate TOMASS performance in clinical populations, particularly individuals with neurological disorders such as Parkinson's disease and dysphagia (Todaro et al. 2021). Additional studies evaluating the influence of oral health variables, including dentition status and prosthesis use, on TOMASS performance are also warranted (Gotfredsen and Walls 2008; Huang et al. 2021).

### 4.1 Conclusion

The Brazilian Portuguese version of TOMASS demonstrated satisfactory reliability for the assessment of solid mastication and swallowing in healthy adults across different age groups. The protocol is objective, standardized, and easy to apply, presenting potential for clinical and research use, representing a significant advance in the assessment of these functions in the Brazilian context.

## Funding

The authors have nothing to report.

## Ethics Statement

The study was approved by the local Research Ethics Committee (approval number 2023‐0402).

## Conflicts of Interest

The authors declare no conflicts of interest.

## Data Availability

The data that support the findings of this study are available from the corresponding author upon request.
